# Surface epithelialization of the type I Boston keratoprosthesis front plate: immunohistochemical and high-definition optical coherence tomography characterization

**DOI:** 10.1007/s00417-012-1960-5

**Published:** 2012-02-28

**Authors:** Lee Kiang, Mark I. Rosenblatt, Rachel Sartaj, Ana G. Alzaga Fernandez, Szilard Kiss, Nathan M. Radcliffe, Donald J. D’Amico, Kimberly C. Sippel

**Affiliations:** 1 Department of Ophthalmology, Weill Cornell Medical College, New York-Presbyterian Hospital, 1305 York Avenue, 12th Floor, New York, NY 10021 USA; 2 The Margaret M. Dyson Vision Research Institute, Weill Cornell Medical College, New York, NY USA

**Keywords:** Keratoprosthesis, Corneal epithelium, Bio-integration

## Abstract

**Background:**

The aim of this work is to characterize a transparent tissue layer partially covering the anterior surface of the type I Boston permanent keratoprosthesis front plate in four patients.

**Methods:**

The tissue over the front plate was easily scrolled back as a single transparent layer using a sponge. In two cases, histopathologic analysis was undertaken and immunofluorescent staining with a cytokeratin 3-specific antibody was performed. The relationship of the tissue to the keratoprosthesis device was further characterized using spectral domain high-definition optical coherence tomography (HD-OCT).

**Results:**

Histopathologic analysis revealed the tissue to be non-keratinized squamous epithelium. No goblet cells were seen, suggesting the cells were of corneal, and not conjunctival, epithelial origin. Immunofluorescent staining of all cells was positive for cytokeratin 3, a protein strongly associated with corneal epithelium. The tissue was easily discerned by HD-OCT and was of substantial thickness near the external junction between the keratoprosthesis device and the carrier corneal tissue. In three cases, visual acuity was unaffected by the presence or absence of this tissue. In one case, a prominent tissue margin temporarily obscured the visual axis and reduced visual acuity; this resolved with mechanical central debridement and has not recurred.

**Conclusions:**

The transparent tissue layer covering the anterior surface of the type I Boston keratoprosthesis front plate was found to represent non-keratinized squamous epithelium, most likely of corneal epithelial origin. This potentially represents a further step in bio-integration of the keratoprosthesis device. In particular, epithelial coverage of the critical junction between the device and the carrier corneal tissue might serve an important barrier function and further reduce the incidence of infection and extrusion of the type I Boston permanent keratoprosthesis.

## Introduction

Permanent keratoprostheses can be sight-restoring devices in patients with severe corneal injury or repeated corneal transplant failure, yet their use has historically been complicated by infection [1, 2] and tissue necrosis [3, 4]. The widely used type I Boston keratoprosthesis [5–13] has a multi-part design consisting of a donor corneal button, serving as a carrier, sandwiched between a plastic polymethyl methacrylate (PMMA) front plate and a PMMA or titanium back plate [10, 14]. The stem connecting the front plate with the back plate provides an optically clear channel for vision. The donor corneal button is sutured into place in standard penetrating keratoplasty fashion.

It has generally been assumed that epithelium does not grow over the plastic front plate of the keratoprosthesis, leaving the junction between the carrier donor corneal button and the keratoprosthesis front plate uncovered by epithelium. Recently, a case of epithelialization of the anterior surface of the keratoprosthesis front plate was reported [15]. Histopathologic analysis in this case demonstrated multilayered, non-keratinized squamous epithelial tissue, presumably of corneal epithelial origin. Our study extends the group of patients in whom this phenomenon has been described to four additional patients, and in addition to histopathologic analysis, we performed immunofluorescent staining of this tissue utilizing a cytokeratin 3 specific antibody. Cytokeratin 3, while having been identified in a number of different tissues, has a known strong propensity for expression in corneal epithelial tissue [16, 17]. Finally, we report the first examination of this tissue in vivo by the use of spectral domain HD-OCT.

## Methods

The patients involved in this retrospective case series were derived from the Cornea Service of the Department of Ophthalmology at Weill Cornell Medical College and were studied after securing approval of the Institutional Review Board. Spectral domain HD-OCT examination of the anterior segment of all patients was performed using the Cirrus instrument (Carl Zeiss Meditec, Jena, Germany). For an HD-OCT control, an unused, identical keratoprosthesis was mounted in a cardboard holder and scanned using the same imaging software.

### Case 1

**Fig. 1:**
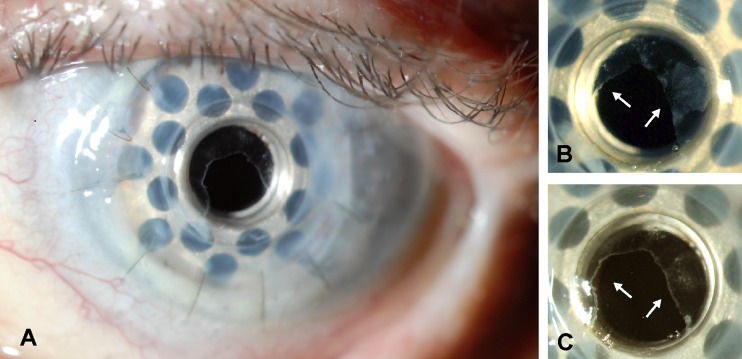
Slit-lamp photographs demonstrating a transparent tissue covering the anterior surface of the front plate of the type I Boston permanent keratoprosthesis. **a**, **b** Case 1, low and higher magnification views. The periphery of the front plate of the keratoprosthesis is covered in its entirety by the tissue (*arrows*), such that the junction between the edge of the keratoprosthesis front plate and the carrier donor corneal button is bridged 360 degrees by the tissue. The center of the keratoprosthesis front plate is not covered by epithelium. **c** One month after removal at the slit lamp with a Merocel™ sponge, the transparent tissue has regrown in a similar configuration

An 87-year-old woman with a history of pseudophakic bullous keratopathy and multiple failed corneal transplants received a type I Boston keratoprosthesis in the left eye, which served to improve her vision from count fingers at 1 foot to 20/70. She was maintained on vancomycin, fluoroquinolone, corticosteroid, and glaucoma drops, and opted against bandage contact lens use. At 8 months postoperatively, a thin transparent tissue was observed covering the periphery of the anterior surface of the front plate (Figs. 1a and b) and this involvement was further detailed by HD-OCT. This tissue was removed several times for the purpose of histopathologic and immunocytopathologic analysis and typically regrew within 1 month (Fig. 1c). Initially, visual acuity was not affected but was later reduced to 20/150 by encroachment of the tissue onto the visual axis. Visual acuity was restored to 20/70 by mechanical debridement of the central epithelium. The tissue has not approached the visual axis since and vision has remained stable for 5 months.

### Case 2

A monocular 71-year-old man with congenital glaucoma and a history of multiple failed corneal transplants underwent type I Boston keratoprosthesis placement in the left eye, which served to improve his vision from hand motion to 20/60. He was maintained on daily vancomycin, fluoroquinolone, and glaucoma drops and intermittently wore a bandage contact lens. Thin transparent tissue was observed over the front plate, covering the periphery of the device, but sparing the visual axis by both slit-lamp and HD-OCT examination, 9 months after placement of the device. Debridement of this tissue for histopathologic and immunocytopathologic analysis did not alter his visual acuity; the tissue regrew within 1 month.

### Case 3

Two months after placement of a type I Boston keratoprosthesis in the left eye, a 69-year-old man with a history of pseudophakic bullous keratopathy, multiple failed corneal transplants, and retinal detachment surgery displayed a clear tissue covering the carrier corneal tissue-device junction, but sparing the visual axis by both slit-lamp and HD-OCT examination. He was on vancomycin, fluoroquinolone, corticosteroid, and glaucoma drops, and was using a bandage contact lens. His vision fluctuated but remained in the range of hand motions, unchanged from before the surgery, due to a retinal detachment that had proved inoperable. Since the visual axis was not affected, the tissue was not debrided.

### Case 4

A monocular 81-year-old woman with chronic angle closure glaucoma and multiple failed corneal transplants underwent placement of a type I Boston keratoprosthesis in the left eye. This served to improve her vision from light perception to 20/50. The patient was lost to follow-up and presented 6 months later exhibiting a transparent tissue over the front plate of the keratoprosthesis. This tissue spared the visual axis by both slit-lamp and HD-OCT examination. The patient had been maintained on a daily regimen of vancomycin, fluoroquinolone, corticosteroid, and glaucoma drops, and a bandage contact lens was in place. The tissue was not debrided since it did not encroach on the visual axis.

### Histopathology and immunohistochemistry

In cases 1 and 2, the tissue over the front plate was scrolled back as a single transparent layer using a Merocel™ sponge (Medtronic, Jacksonville, FL). Tissue samples were submitted in CytoLyt™ solution (Cytyc Corporation, Marlborough, MA), fixed to glass slides, and stained with hematoxylin and eosin (H&E). Indirect immunofluorescence for cytokeratin 3 was performed using mouse anti-keratin K3 monoclonal primary antibody (Millipore, Billerica, MA) and goat anti-mouse Alexa Fluor 488 secondary antibody (Invitrogen, Carlsbad, CA). The DNA stain propidium iodide (Invitrogen, Carlsbad, CA) was used to identify cell nuclei, facilitating visualization of cells and allowing for assessment of the percentage of the cell population expressing cytokeratin 3.

## Results

**Fig. 2:**
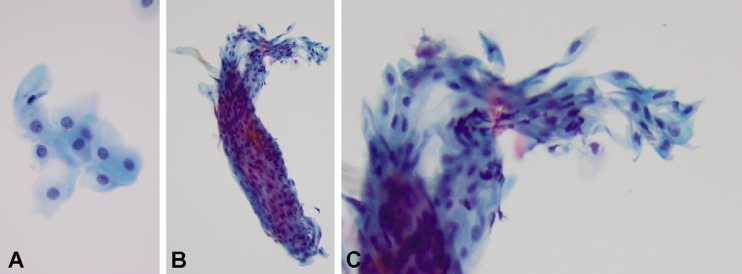
Hematoxylin and eosin (H&E) stain of tissue removed in cases 1 and 2. **a** Tissue removed in case 1 consists of non-keratinized squamous epithelial cells without evidence of dysplasia. **b**, **c** Low and higher magnification views of a cohesive scroll of similar tissue removed in case 2

**Fig. 3:**
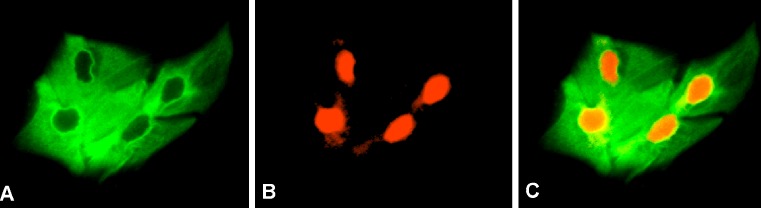
Indirect immunofluorescence analysis of tissue removed in case 2. **a** Antibody staining (*green color*) demonstrates the tissue expresses cytokeratin 3, a protein typically associated with corneal epithelium. **b** Propidium iodide nuclear stain (*red color*) is used to identify cell nuclei. **c** Overlay of cytokeratin 3 antibody and propidium iodide nuclear stains demonstrates all cells express cytokeratin 3

Light microscopic analysis of the tissue in case 1 revealed the tissue to be non-keratinized squamous epithelium without evidence of dysplasia (Fig. 2a). No goblet cells were seen, suggesting the cells were of corneal, and not conjunctival, epithelial origin. In all cells (nuclei marked by propidium iodide) immunofluorescent staining was positive for cytokeratin 3, a protein strongly associated with corneal epithelium. Analysis of the tissue in case 2 similarly revealed non-keratinized squamous epithelium without goblet cells (Fig. 2b, c) in which all cells stained positively for cytokeratin 3 (Fig. 3).

**Fig. 4:**
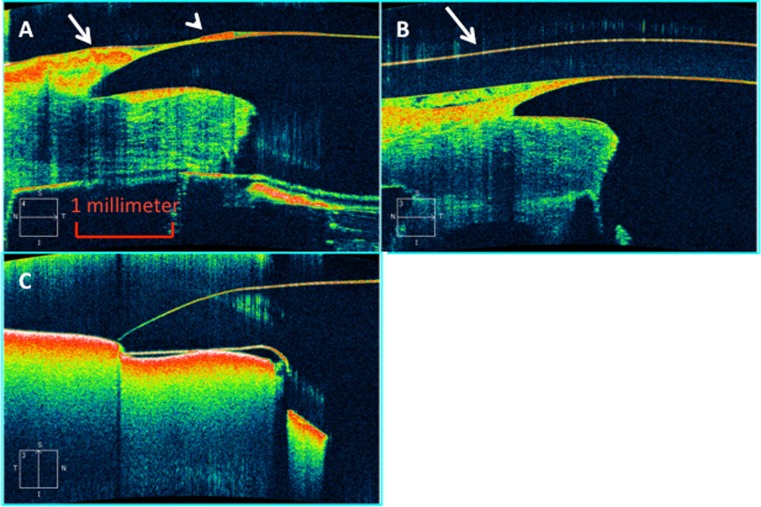
Spectral domain HD-OCT examination of cases 1 and 4. **a** Case 1. There is a focally prominent collection of surface tissue (*long arrow*) overlying the carrier corneal tissue-keratoprosthesis junction which continues onto the front plate with a second smaller collection (*arrowhead*) before thinning and ending to leave the front plate uncovered in its center. **b** A similar collection of tissue is evident in case 4. The* arrow* identifies a Kontur bandage contact lens (Kontur Kontact Lens Co., Inc., Hercules, CA) overlying the keratoprosthesis. **c** An unused type-I Boston keratoprosthesis mounted in a cardboard box for comparison

HD-OCT examination was readily able to detect this surface tissue and delimit its extent (Fig. 4). In particular, in every patient examined there was substantial prominence of the tissue at the junction between the carrier corneal tissue and the keratoprosthesis, with variable extension of a thinner layer of tissue onto the front plate of the device.

## Discussion

These four described cases provide evidence of the growth of a regenerating, non-keratinized squamous epithelium over the surface of the PMMA front plate of the type I Boston permanent keratoprosthesis. Histopathologic and immunofluorescence analysis performed in two of the cases indicate the probable corneal epithelial origin of this tissue with the host limbus presumably serving as the source of these cells. Two of the patients did not regularly wear a bandage contact lens, suggesting that the tissue was sufficiently adherent to the PMMA to withstand the wiper action of the eyelids.

Epithelialization of the plastic front plate of the Boston keratoprosthesis is of clinical significance in that it potentially represents a further step towards the desired goal of bio-integration of the device [18]. Current recommendations for Boston keratoprosthesis patients include prophylactic topical antibiotics for life to minimize the risk of endophthalmitis [2, 14, 19, 20]. An intact epithelial layer covering the junction between the carrier donor corneal tissue and the edge of the keratoprosthesis front plate in its entire circumference, as seen in our patients, would presumably decrease the infection risk by constituting a barrier for entry of microorganisms [21]. Moreover, tissue necrosis adjacent to the keratoprosthesis stem may relate to the proteolysis of corneal stromal tissue by enzymes in the tear film; epithelium spanning the prosthesis-corneal tissue interface would presumably again serve a beneficial barrier function.

What may have allowed epithelium to grow over the keratoprosthesis front plate? Generally, epithelial cells require a basement membrane or positively charged surface on which to adhere and migrate. Epithelial cells have been cultured on substrates such as amniotic membrane and silk, as well as specially treated plastic. A recent study in rabbits using a keratoprosthesis with a silicone optical core suggests that coating the prosthesis with type I collagen improves epithelial cell adherence and migration [22]. Our patients were not distinguished by race, gender, age, perioperative medications, or underlying ophthalmic diagnosis as compared to other keratoprosthesis patients. Two of the patients routinely wore a soft bandage contact lens and two did not. Of possible significance is that all four patients received the most recently developed “threadless” model of the Boston type I keratoprosthesis which, in addition to other modifications, exhibits a smaller 5-mm-diameter front plate compared to previous 6–7-mm diameter designs, although our study cannot determine if the smaller size is responsible.

Khalifa and associates [15] in their case report of one patient manifesting epithelialization of the anterior surface of the Boston keratoprosthesis front plate indicate the epithelial tissue significantly interfered with vision, decreasing vision from 20/70 to 20/400. This decline in vision was attributed to a lack of uniformity in the thickness of the epithelial tissue. In our series of patients, with one exception, the epithelium did not appear to affect visual acuity, probably because the central part of the keratoprosthesis front plate was not involved. Given that the epithelium was potentially of benefit, our management approach was to leave the epithelium in place. The exception was one patient in whom the epithelium at one point did encroach on the visual axis. The vision was restored to baseline after mechanical debridement of the central epithelium; the prosthesis-corneal tissue interface was left covered by epithelium.

The surface epithelialization of the front plate of the type I Boston keratoprosthesis may prove not to be uncommon, in particular in the new threadless design, although the precise percentage of patients in whom this phenomenon occurs spontaneously requires further elucidation. In our view, epithelial coverage, at least of the junction between keratoprosthesis and carrier donor corneal tissue, and further bio-integration without limitation of vision would be a desired goal with the type I Boston keratoprosthesis. Further studies will be needed to understand the mechanism by which epithelial growth over prosthetic material occurs such that advances in keratoprosthesis composition, design, and surface modifications may be used to optimize the process.
